# Physiological Responses in Trail Runners during a Maximal Test with Different Weighted-Vest Loads

**DOI:** 10.3390/sports12070189

**Published:** 2024-07-11

**Authors:** Germán Jiménez-Redondo, Bruno Castro-Frecha, Francisco Javier Martínez-Noguera, Pedro E. Alcaraz, Cristian Marín-Pagán

**Affiliations:** Research Center for High-Performance Sport, Campus de los Jerónimos, Catholic University of Murcia, Guadalupe, 30107 Murcia, Spain; gjimenez9@alu.ucam.edu (G.J.-R.); bcastro8@alu.ucam.edu (B.C.-F.); palcaraz@ucam.edu (P.E.A.); cmarin@ucam.edu (C.M.-P.)

**Keywords:** endurance, metabolism, overload, lactate, threshold, oxygen uptake

## Abstract

During some trail running races, athletes have to carry hydration support, food, and technical and safety equipment, which generates an additional load that must be mobilized during the race. The aim of the present study was to determine the physiological responses to overload running and the effect they may have on metabolic zones. Seventeen well-trained male trail runners (n = 17) completed three maximal treadmill tests with weighted vests at 0%, 5%, and 10% of their body mass (L0, L5, and L10). Their gas exchange was monitored to assess their ventilatory thresholds 1 (VT1) and 2 (VT2), maximal fat oxidation zone (FatMax), and peak oxygen consumption (VO_2peak_). Their heart rate (HR), power, and velocity (V) were tracked to compare their behavior. One-way ANOVA showed significant differences in the V (*p* < 0.001; ηp2 = 0.4620) as a limitation for reaching the peak velocity (V_peak_), with a significant decrease in the V_peak_ with the L10 compared to the L0 (*p* = 0.002) and L5 (*p* = 0.004). In addition, one-way ANOVA showed significant differences in the peak absolute power (*p* < 0.001; ηp2 = 0.468) among the groups, detecting higher power production between the L10 and L0 (*p* < 0.001) and between the L10 and L5 (*p* = 0.015). Loads higher than L5 could generated important physiological and mechanical modifications, while a load of L5 managed to maintain the working conditions without overloading. These insights shed light on nuanced strategies for optimizing performance and endurance, offering valuable considerations for athletes seeking to enhance their training regimens during overload conditions.

## 1. Introduction

Trail running (TR) races are characterized by predominantly natural terrain (up to 75%) and do not necessitate orienteering skills for the participants. This unique blend of attributes has fueled a notable surge in both participation rates and the professionalization of TR as a sport [1,2]. Additionally, TR encompasses a diverse range of formats, spanning from shorter events, like vertical kilometers (1000 m elevation, where the incline is not less than 25%), to longer mountain ultra-marathons exceeding traditional marathon lengths [3].

Performance in TR is influenced by factors such as the high aerobic power (VO_2max_) and optimized lactate thresholds, as well as low body fat mass [4,5,6,7]. Metabolic demands and performance determinants in TR differ from those in road running [4,8,9,10], primarily due to the varying terrain gradients encountered, which elicit different biomechanical, neuromuscular, and metabolic adaptations [11]. Uphill running (UR) typically involves shorter strides, longer ground contact times, and forefoot striking, whereas downhill running (DR) is characterized by longer strides, shorter ground contact times, and rearfoot striking [12] UR relies more on concentric muscle actions, while DR necessitates eccentric muscle actions [13], resulting in differing degrees of muscle damage and energy expenditure (EE) [11,14,15,16,17]. Increases in creatine kinase, creatinine, blood urea nitrogen, and albumin have been observed after a 35 km trail run (+1815 m of elevation gain) [18]. In addition, this effect has also been found after finishing a marathon, which has less elevation gain but a similar distance to some trail running competitions [19].

Given the endurance nature of TR, athletes often require nutritional and hydration support during longer races [20], with many opting to self-carry provisions, adding load that must be accounted for in training strategies. Therefore, resistance training methods can be considered for inclusion in the training plans of TR athletes. In this regard, resistance running methods, including sleds, parachutes, and loaded running (LR) with vests, have been extensively studied for their efficacy in developing speed and neuromuscular capabilities [21,22,23,24]. Higher loads with vests during sprinting have been shown to impact the maximal speed and flight time, increasing the ground contact time [25]. Similarly, submaximal velocities with added loads result in shorter flight times and greater ground contact [26].

However, there is limited evidence regarding the metabolic effects of loaded walking or LR. Some studies have suggested increased metabolic demands, including elevated oxygen consumption (VO_2_), heart rate (HR), and EE when walking with added load on inclines [27,28]. Conversely, increased loads at a fixed slope may also elevate metabolic demands during walking [29]. With LR, greater carbohydrate utilization has been observed compared to unloaded running, along with elevated blood lactate levels [27,30]. However, the effects on other physiological and performance parameters remain unexplored. Therefore, the evaluation of the effect of loading on the shift of carbohydrate and fat oxidation is essential to be able to optimally adjust nutrient intake during trail running competitions.

Previous results [31] showed significant interactions between different overloads and performance during a walking test. Notably, differences in the VO_2_ were observed between the 0% of body mass (BM) condition and all other weighted-vest (WV) conditions, as well as between the 10% and 20% BM conditions across all walking speeds. Additionally, disparities in the relative exercise intensity were found at higher speeds but not at slower speeds. These findings contribute to our understanding of the metabolic and biomechanical implications of loaded walking, highlighting the impact of varying WV conditions on physiological responses and performance measures. In this sense, and taking into account that extra load can generate metabolic and biomechanical changes in walking, it is necessary to evaluate how extra load can affect the metabolic, mechanical, and performance variables in trail runners.

This study aimed to examine the physiological and metabolic differences induced by overloads in trail runners during a maximal incremental metabolic test to know how overloading could affect the metabolic zones expression (thresholds, FatMax, VO_2peak_). We hypothesized that added load could modify the physiological responses to the same intensity, shifting the metabolic thresholds and peak values in an incremental test and anticipating fatigue (loss of performance). This will be of great importance for coaches’ training prescriptions.

## 2. Materials and Methods

### 2.1. Experimental Design

The current investigation was structured as a cross-over study and received approval from the Ethics Committee at the Catholic University of Murcia (CE012104). All protocols adhered to the ethical guidelines outlined in the World Medical Association’s Declaration of Helsinki (2013) [32]. The athletes underwent three maximal tests utilizing varying loads: 0% (L0), 5% (L5), and 10% (L10) of their BM, applied by a WV, enabling the analysis of physiological disparities.

### 2.2. Participants

Seventeen well-trained male trail runners participated in this study (Table 1). All participants met the following inclusion criteria: (i) male, between 18 and 40 years old; (ii) no injuries that hindered training for more than 1 week in the preceding 4 months; (iii) engagement in at least 4 training sessions weekly; (iv) a minimum of 1 year of experience in trail running; (v) abstention from consuming any supplements for 2 weeks preceding the study; (vi) the absence of any pathological conditions. Before participation, all individuals were briefed on the study protocols and provided informed consent by signing the appropriate documentation. Using a medium effect size (f = 0.50), a sample size of 34 participants was considered sufficient to provide 80% power to detect intergroup interactions (0%, 5%, and 10%), with the probability of error value α set at 0.05 (G*Power, version 3.0.10). To account for the possible loss of subjects, we increased the target sample size to 51 participants.

### 2.3. Procedures

**Figure 1:**
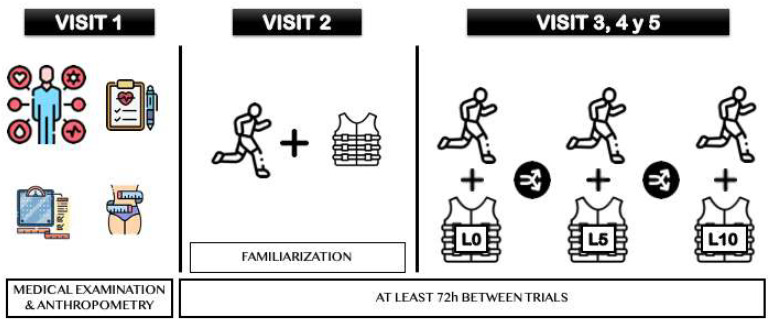
Protocol for visits 1, 2, 3, 4 and 5, for intervention groups with weighted vests of 0%, 5% and 10% of their body mass. L0 = 0% body mass overload; L5 = 5% body mass overload; L10 = 10% body mass overload.

The participants attended the laboratory 5 times, with each visit separated by at least 6 days (Figure 1). The initial visit served as an orientation to the procedures and medical approval, while the subsequent visits were dedicated to conducting maximal incremental tests utilizing a metabolic cart. Throughout all sessions, the athletes adhered to a prescribed diet and refrained from training in the 24 h preceding each visit. Visit 1: The athletes completed a medical exam, including a resting electrocardiogram and medical exploration. Anthropometric measurements were conducted according to the International Society for the Advancement of Kinanthropometry (ISAK) guidelines [33] during this first visit. The fat mass percentage was subsequently determined using the Yuhasz equation [34]. Visits 2, 3, 4, and 5: The first trial (visit 2) served as a familiarization session with the maximal incremental test and utilized a randomized load (L0, L5, or L10). Randomization was facilitated through software (Randomizer 4.0, Lancaster, PA, USA) to assign codes to the study groups generated [35]. The subsequent trials involved the completion of each load in a randomized order. The average load for the L5 was 3.55 kg, and it was 7.10 kg for the L10. All trials were conducted at consistent times of the day. For the trials described as conducted with an extra L0 of body mass (BM), the participants wore a weighted vest (330 g) without additional load. The days before visits 3, 4, and 5, the athletes adhered to a standardized diet consisting of 9.0 g/BM carbohydrate, 1.5 g/BM protein, and 0.9 g/BM fat. Additionally, the participants were instructed to consume a standardized breakfast 2 h before the test, comprising 1.3 g/BM carbohydrate, 0.43 g/BM protein, and 0.57 g/BM fat. The meals the day before and the breakfasts before the tests were prepared by a sports nutritionist and sent to the participants 2 weeks before the start of the study. In the previous 24 h, no training was to be performed, and in the previous 72 h, only low-moderate intensity training was allowed.

### 2.4. Tests

#### 2.4.1. Incremental Test

**Figure 2:**
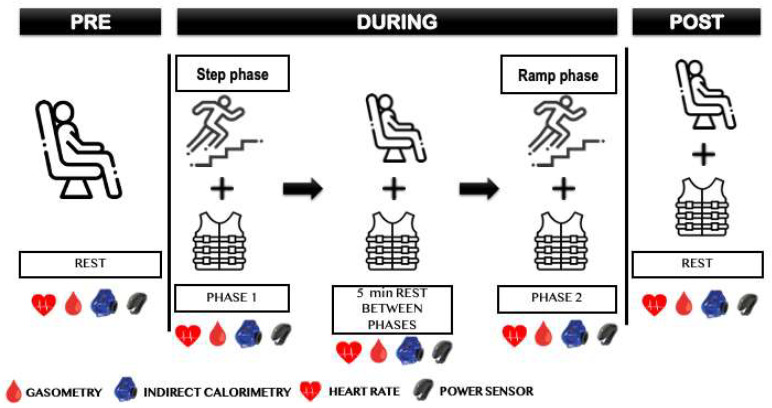
Performance tests and measurements to be carried out in the different phases of the exercise protocol.

A maximal incremental metabolic test was conducted using a two-phase protocol (Figure 2) on a treadmill (Trackmaster treadmill, Newton, KS, USA). This approach combined the assessment of the VT1 and VT2 through a step phase, followed by a ramp phase, to determine the peak values [36,37,38]. Before starting, the blood lactate, SO_2_, and pH levels were evaluated to stablish the resting conditions. The step phase started at 5 km/h, increasing by 1 km/h every 2 min. This first phase concluded when the respiratory exchange ratio (RER) reached a value of 1 for more than 30 s within the same step. At this juncture, the athletes reported their perceived exertion on a modified Borg Scale (RPE) from 1 to 10 [39]. Subsequently, the participants rested for 5 min to mitigate fatigue associated with the first phase [40]. The second phase started at the final velocity of the first phase and increased by 1.5 km/h every minute as a final ramp (0.25 km/h every 10 s), culminating in exhaustion. The athletes reported their perceived exertion using the modified Borg Scale (RPE) at this point, and we extracted blood samples to evaluate their blood lactate, SO_2_, and pH levels in both testing phases (step and ramp).

The gas exchange was monitored using a metabolic cart (Cortex Metalyzer 3B. Leipzig, Germany). The collected data were averaged every 30 s to compute all variables. The VT1 was determined utilizing the ventilatory equivalent method described by Wasserman [41]. The VT2 was identified when the RER reached a value of 1.00 for more than 30 s. The FatMax, representing the percentage of VO_2_ where maximal fat oxidation occurs, was calculated using the Frayn equation [42]. The running economy (RE) was evaluated as the volume of oxygen consumed per BM per kilometer (mL·kg^−1^·km^−1^).

#### 2.4.2. Power Sensor and HR

The Stryd power sensor (Stryd 1, Boulder, CO, USA) was affixed to the top of each athlete’s shoe to measure the power output during all trials. Also, the HR was assessed by the Polar H10 chest band connected to the Polar Flow app (Polar, Kempele, Finland).

#### 2.4.3. Blood Gas Analysis

The resting blood lactate, pH, and oxygen saturation (SO_2_) levels were analyzed in each test using a gasometer (ABL90 flex, Radiometer, Copenhagen, Denmark). This procedure was repeated before the initial test, after each maximal incremental test to evaluate the effort exerted, and at the finish of the ramp phase during each trial [39,43] (Figure 2). Blood samples were obtained via capillary puncture, with a sample volume of 65 microliters, by an experienced technician.

### 2.5. Statistical Analysis

Statistical analysis was conducted utilizing Jamovi 2.3.18 (Jamovi, Sydney, Australia), and all descriptive statistics were reported as the mean ± standard deviation (SD). The homogeneity and normality of the data were assessed using Levene’s and the Shapiro–Wilk tests, respectively. The variables at the FatMax, VT1, VT2, and peak values, such as the HR, RER, VO_2_R_peak_, VO_2peak_, V, AbsPw, Pw/BM, trial duration, and values from the step and ramp phases, were analyzed employing one-way ANOVA. However, the lactate, pH and SO_2_ variables were analyzed by two-way ANOVA with repeated measures, a time factor (pre vs. post), and a condition factor (L0 vs. L5 vs. L10). Tukey’s post hoc analysis was performed if statistical significance was observed in the ANOVA models. Trials conducted at L0 served as the reference point. Statistical significance was defined as *p* = ≤0.05, while *p* = <0.10 indicated a trend toward statistical significance. Partial eta square (ηp2) values were interpreted as follows: <0.01 (irrelevant), ≥0.01 (small effect), ≥0.059 (moderate effect), and ≥0.138 (large effect) [44].

## 3. Results

### 3.1. Peak Values

**Figure 3:**
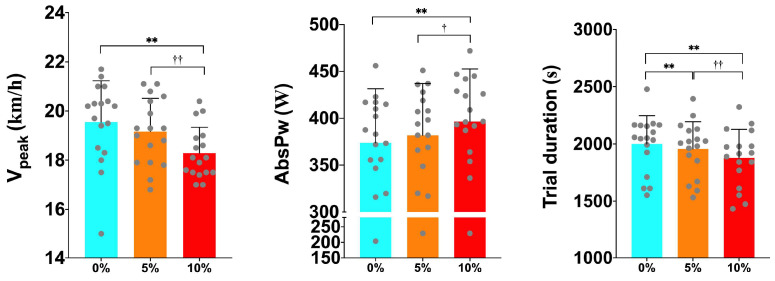
Differences in peak velocity, absolute power, and trial duration during the exercise protocol among the different intervention groups (0%, 5%, and 10%). AbsPw = absolute power output L0 = 0% body mass load; L5 = 5% body mass load; L10 = 10% body mass load; ** = significant difference with respect to 0%; † = trend with respect to 5%; †† = significant difference with respect to 5%.

Table 2 shows HR values, metabolic–biomechanical variables, and trail duration. ANOVA found no significant differences for the relative VO_2_R_peak_ (*p* = 0.145) and absolute VO_2peak_ (*p* = 0.342), however, we detected significant changes in the trial duration (*p* = ≤0.001; ηp2 = 0.644) when performing the tests with different loads. Tukey’s post hoc revealed longer trial durations for the L0 trials compared to the L10 (*p* = ≤0.001), and for the L5 compared to the L10 (*p* = ≤0.001) (Table 2, Figure 3). In addition, we also observed significant differences in the peak velocity (V_peak_) (*p* = <0.001; ηp2 = 0.462). Tukey’s post hoc analysis showed changes between the L0 and L10, (*p* = 0.002) and between the L5 and L10 (*p* = 0.004) (Table 2, Figure 3). Concerning the absolute power (AbsPw), we detected significant differences among the groups (*p* = ≤0.001; ηp2 = 0.468) (Table 2). Tukey’s post hoc showed that the L10 produced more W than the L0 (*p* = <0.001) and L5 (*p* = 0.020) (Table 2, Figure 3). The results observed or the V_peak_, power, and trial duration are in line with our stated hypothesis; increased load produced a negative effect on the mechanical expression and performance variables, but not on the relative or absolute VO_2peak_.

### 3.2. VT1

**Figure 4:**
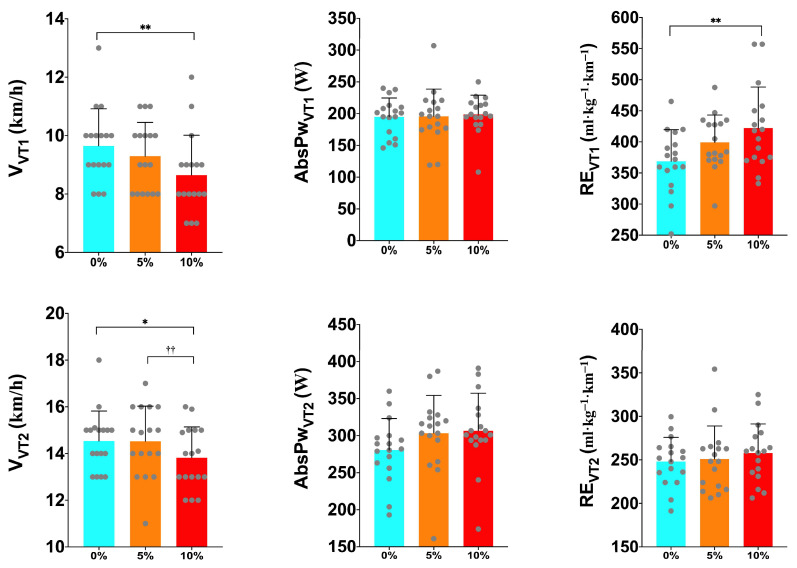
Differences in velocity, absolute power, and running economy in VT1 and VT2 during the exercise protocol among the different intervention groups (0%, 5%, and 10%). AbsPw = absolute power output; L0 = 0% body mass load; L5 = 5% body mass load; L10 = 10% body mass load; RE= running economy; V = velocity; VT1 = ventilatory threshold 1; VT2 = ventilatory threshold 2; * = trend with respect to 0%; ** = significant difference with respect to 0%; †† = significant difference with respect to 5%.

Table 3 shows the HR values and metabolic–biomechanical variables measured at VT1 and VT2. ANOVA found significant differences for the physiological parameters: V_VT1_ (*p* = 0.015; ηp2 = 0.230) and RE (*p* = 0.009; ηp2 = 0.253) (Table 3, Figure 4). Tukey’s post hoc found that the L0 achieved a higher V_VT1_ than the L10 (*p* = 0.041). In addition, Tukey’s post hoc detected that the RE was lower in the L0 compared to the L10 (*p* = 0.024) (Table 3, Figure 4).

### 3.3. VT2

ANOVA detected significant interactions among the groups in the V (*p* = 0.041; ηp2 = 0.180) in the VT2. Tukey’s post hoc found a trend between the L0 and L10 (*p* = 0.063) and a significant difference between the L5 and L10 (*p* = 0.014) in the V that was reached in the VT2 (Table 3, Figure 4). Furthermore, ANOVA found significant interactions among the groups in AbsPW (*p* = 0.036; ηp2 = 0.242) in the VT2 (Figure 4). In contrast, Tukey’s post hoc found no significant differences among the groups. Moreover, ANOVA found no significant interactions among the groups for the HR (*p* = 0.106; ηp2 = 0.274), relative power (*p* = 0.224; ηp2 = 0.109), and %VO_2peak_ (*p* = 0.161; ηp2 = 0.456) in the VT2 (Table 3). The results found in the VT1 are in line with the established hypothesis; metabolic (RE) and mechanical (V) changes were found among the groups, with only biomechanical changes in the VT2.

### 3.4. FatMax

Table 4 shows the HR values and metabolic–biomechanical variables measured at the FatMax. ANOVA found no significant interactions among the groups for all variables analyzed at the FatMax (Table 4).

### 3.5. Blood Lactate, pH, sO_2_, and RPE

Table 5 shows the values of the capillary blood biochemical markers analyzed pre- and post-exercise protocol. The results of the two-way ANOVA repeated measures detected no significant differences in the time × group interaction for SO_2_ (*p* = 0.785; ηp2 = 0.015). However, a trend was observed in the time × group interaction with a large effect size for the pH levels (*p* = 0.083; ηp2 = 0.144) (Table 5). In addition, Tukey’s post hoc detected significant differences in the pH in the between-group comparison (*p* = 0.031).

On the other hand, the two-way ANOVA repeated measures found no time × group interaction in the lactate (*p* = 0.309; ηp2 = 0.071). The maximum lactate value obtained was 11.58 mmol/L at load L5, which represents the mean maximum value. Moreover, the L0 and L10 loads accounted for 99.13% and 94.9% of the mean maximum, respectively.

## 4. Discussion

The present study aimed to determine possible differences in metabolic zones, such as the VT1, VT2, VO_2max_, FatMax, performance, and capillary blood biochemical markers, during a maximal test with different additional loads (L0, L5, and L10 of BM). The findings of this study shed light on the physiological and metabolic implications of running with additional loads, particularly in trail runners. Through comprehensive analysis, significant differences in key performance metrics, such as velocity, power, and ventilatory thresholds, have been revealed across varying load conditions. These insights not only deepen our understanding of the impact of added loads on running performance but also offer practical implications for athletes and coaches seeking to optimize training strategies in trail running environments. Our initial hypothesis has been partially fulfilled, as a significant negative effect on biomechanical variables was observed with L10, but in the L5 group, no significant loss of performance was observed.

Regarding the peak values, only the mechanical variables showed significant differences. The V_peak_ for the L10 group was 6.64% lower than for the L0, while the AbsPw showed higher values with the L5 and L10 loads with respect to the L0. There is only one other study that has investigated the effects of running at speeds close to the maximum VO_2_ with WV [26]. However, it focused on other biomechanical variables, such as the flight and contact time, frequency, and stride length, so a direct comparison cannot be made. The V_peak_ in a maximal incremental test is a crucial variable for performance control and race time prediction in TR [4]. Therefore, it is important to consider the overload in maximal work, especially if training by power parameters.

On the other hand, the VT1 was assessed at intensities between 55% and 57% of the VO_2peak_. Gaffney et al. [27] used intensity within that range (55% VO_2max_) to observe physiological differences in a 30 min run in CrossFit^®^ practitioners. Gaffney et al. shower significant differences in the VO_2_ (+0.22 L·min^−1^ for men; +0.07 L·min^−1^ for women), HR (7% for both groups), and RER (+0.04 for men; +0.02 for women) with increasing loads [27]. However, in our study, the %VO_2peak_ at which the VT1 occurred was not different among the different loads. Furthermore, looking at the increase in the RER values experienced by the CrossFit^®^ practitioners with higher loads [27], the same increase could be expected for TR athletes when running with additional load. The observed differences compared to the existing evidence [27] could be due to the weight of the added load, test duration, and participant characteristics (sport specialty and gender). Gaffney et al. [27] used a higher load for men (12.99% BM) than the maximal load of our study (L10) and a similar load for women (9.94% BM). Their test also lasted longer, with the participants running at a fixed speed for 30 min, while our athletes completed an incremental maximal test. On the contrary, the RER did not show significant differences. The RER is a parameter of interest in submaximal intensities (below the VT2) because changes in this variable reflect bioenergetic behavior, showing different contributions of macronutrients (fat and carbohydrates).

After analyzing the data in the VT2, we observed that this threshold was between 82% and 85% of the VO_2peak_, and we found no significant differences in the metabolic parameters among the groups. However, we did find significant differences in the V in the VT2. These differences could be due to the characteristics of the sample, as TR practitioners may be lighter (71.0 ± 9.9 kg vs. 78.12 ± 10.9 kg) and could be more adapted to this load. An explanation of this result could be a greater contribution of the elastic component of the muscle–tendon system when running with L5. The running economy showed differences at the VT1 point due to a potentiation warm-up [45] and, more recently, an intra-trial effect [46] was described. Cartón-Llorente et al. [46] used similar loads to those used in the present study and showed higher leg spring stiffness for the L5 than for the L0 and L10. A second explanation for why this has only been observed in trail runners could be a higher load limit. In other cases, this limit could be reached by an increased BM or lack of specific strength and adaptation.

No significant differences were found in the FatMax, probably due to the small sample size for the variability that this parameter can show, despite controlling for previous diets and comparing the same athletes in the three conditions. Although Purdom et al. [30] studied fat oxidation at different intensities, they did not determine the FatMax. Additionally, no value reported any statistical difference at this point. It will be necessary to further study this parameter and to design specific studies to monitor bioenergetics during sustained and prolonged efforts.

Unlike previous evidence that described an increase in blood lactate (+0.6 mmol/L, *p* < 0.05) associated with loaded running [27], the final blood lactate levels in this study showed no differences. However, this could be because, despite matching the speed, the participants in the other study exerted efforts at different intensities in different tests. On the other hand, our trail runners reached almost the maximum intensity with each load.

Lastly, the results of different studies could support the idea that greater changes in different variables might occur in UR. It has been shown how a positive slope results in greater concentric load and greater EE [11], while BMI has been proven to be a predictor of UR performance [4]. The mass of the vest would artificially increase the BMI, while the predominance of concentric contraction would eliminate the hypothetical increased stiffness that a certain load might provide in level running [46]. Additionally, an increase in load has been used in previous studies [29] to raise metabolic intensity in uphill walking.

In this study, we aimed to make a first approximation in a laboratory test, which is the most common method for the evaluation of metabolic zones and the most stable one to see possible differences with overloads. However, the nature of this study brings some limitations. Trail running occurs across terrains characterized by numerous changes in gradient, alternating between UR and DR, a phenomenon not addressed in this study. Our study has the limitation of being carried out on a very specific sample (well-trained male athletes), so caution should be exercised when extrapolating the results to another type of sample (women, elite athletes, etc.). These findings represent an inaugural attempt to juxtapose physiological changes associated with L0, L5, and L10 additional loads with a weighted vest during treadmill running. Based on our results, future research is needed to evaluate the effect of a weighted vest in a rectangular test (submaximal intensity and long duration) and how it may affect the oxidation of different energy substrates.

## 5. Conclusions

Our study presents novel data on a topic that is under-researched and generates some uncertainty in coaches and athletes about how to control training loads when training or competing with additional load. This investigation offers valuable insights for sports scientists aiming to refine training load adjustments and competition monitoring strategies based on the level of overload experienced by athletes. In summary, our findings indicate that 5% extra load does not induce notable physiological or mechanical shifts, contrasting with the discernible alterations triggered by 10% extra load. Notably, 10% extra load may lead to changes, such as the displacement of the VT2 and a reduction in the velocity parameters (such as the running rhythm). Therefore, coaches and athletes should take into consideration the adjustment of intensity zones when working with loads greater than 5% of one’s body mass. The results found in this study provide valuable information for coaches to be able to establish more precise criteria when structuring training plans according to the loads that their athletes will be carrying in competitions.

## Figures and Tables

**Table 1:** General participant characteristics (n = 17).

Age (Year)	Weight (kg)	Height (cm)	Distance/Week (kms)	Elevation Gain/Week	Fat Mass (%)
31.08 (6.45)	71.02 (9.95)	178.77 (7.88)	55.23 (22.44)	1276.93 (683.31)	7.48 (0.99)

**Table 2:** Differences in the physiological and biomechanical parameters at peak values during the exercise protocol in trail runners.

Variables	L0	L5	L10	*p*	ηp2
HR (bpm)	183.3 (9.3)	186.08 (9.20)	187.2 (9.7)	0.800	0.027
RER	1.12 (0.50)	1.11 (0.60)	1.11 (0.50)	0.599	0.031
VO_2_R_peak_ (mL·kg^−1^·min^−1^)	63.3 (5.9)	62.6 (6.8)	62.2 (7.8)	0.342	0.065
VO_2peak_(L/min)	4.3 (0.5)	4.3 (0.4)	4.2 (0.4)	0.145	0.114
V_peak_ (km/h)	19.6 (1.7)	19.20 (1.35)	18.3 (1.1) **, ††	<0.001 ##	0.462
AbsPw (W)	373.8 (59.7)	381.9 (59.1)	396.6 (57.8) **, †	0.003 ##	0.468
Pw/BM (W/kg)	5.4 (0.4)	5.3 (0.4)	5.4 (0.5)	0.149	0136
Trial duration (s)	2000.1 (247.0)	1956.8 (237.8) **	1878.4 (249.3) **, ††	<0.001 ##	0.644

Peak values; HR = heart rate; RER = respiratory exchange ratio; VO_2_R_peak_ = absolute maximum oxygen consumption; VO_2peak_ = relative maximum oxygen consumption; V_peak_ = velocity at VO_2peak_; AbsPw = absolute power; Pw/BM = relative to body mass power; L0 = 0% body mass load, L5 = 5% body mass load; L10 = 10% body mass load; ## = significant difference ANOVA; †: trend with respect to 5%; ** = significant difference with respect to 0%; †† = significant difference with respect to 5%. ηp2 = partial eta square; *p* = ANOVA between-group differences.

**Table 3:** Differences in the physiological and biomechanical parameters of VT1 and VT2 during the exercise protocol in trail runners.

Zone	Variables	L0	L5	L10	*p*	ηp2
VT1	HR (bpm)	123.7 (11.7)	129.0 (14.7)	119.8 (16.4)	0.301	0.139
	RER	0.90 (0.04)	0.90 (0.03)	0.90 (0.04)	0.972	0.002
	% VO_2peak_	54.5 (5.9)	56.7 (5.0)	54.8 (5.0)	0.456	0.048
	V (km/h)	9.7 (1.3)	9.3 (1.2)	8.7 (1.4) **	0.015 ##	0.230
	AbsPw (W)	195.0 (30.5)	195.8 (45.8)	198.9 (31.1)	0.605	0.038
	Pw/BM (W/kg)	3.5 (0.7)	3.86 (0.60)	3.72 (0.50)	0.135	0.153
	RE (mL·kg^−1^·km^−1^)	368.8 (50.7)	399.1 (44.0)	422.1 (65.9) **	0.009 ##	0.253
VT2	HR (bpm)	169.0 (9.6)	172.3 (9.7)	173.4 (12.9)	0.106	0.274
	% VO_2peak_	82.2 (7.4)	85.7 (6.3)	84.7 (6.7)	0.182	0.101
	V (km/h)	14.5 (1.3)	14.5 (1.5)	13.8 (1.3) *, ††	0.041 ##	0.180
	AbsPw (W)	280.4 (44.0)	303.3 (54.5)	306.7 (54.0)	0.035 ##	0.242
	Pw/BM (W/kg)	4.8 (0.9)	5.0 (0.5)	4.9 (0.5)	0.224	0.109
	RE (mL·kg^−1^·km^−1^)	248.0 (27.9)	250.9 (37.9)	257.8 (33.5)	0.352	0.063

VT1 = ventilatory threshold 1; VT2 = ventilatory threshold 2; HR = heart rate; RER = respiratory exchange ratio; %VO_2peak_ = VO_2_R_peak_ = peak of oxygen consumption normalized to body weight; V = velocity; AbsPw = absolute power output; Pw/BM = power output normalized to body weight; RE = running economy; L0 = 0% body mass load, L5 = 5% body mass load; L10 = 10% body mass load; ## = significant difference ANOVA; * = trend with respect to 0%; ** = significant difference with respect to 0%; †† = significant difference with respect to 5%. ηp2 = partial eta square; *p* = ANOVA between-group differences.

**Table 4:** Differences in the physiological and biomechanical parameters in the FatMax during the exercise protocol in trail runners.

Variables	L0	L5	L10	*p*	ηp2
HR (bpm)	98.1 (13.9)	101.2 (17.3)	99 (14.2)	0.812	0.023
RER	0.82 (0.10)	0.82 (0.10)	0.83 (0.10)	0.718	0.020
% VO_2peak_	38.4 (12.4)	41.8 (14.2)	37.9 (10.3)	0.597	0.032
V (km/h)	6.8 (1.6)	7.2 (1.9)	6.5 (1.6)	0.462	0.047
AbsPw (W)	125.9 (41.6)	144.8 (70.3)	132.8 (44.7)	0.377	0.072
Pw/BM (W/kg)	2.4 (0.7)	2.5 (0.7)	2.4 (0.6)	0.719	0.025
RE (mL·kg^−1^·km^−1^)	472.8 (197.7)	450.6 (184.0)	498.2 (18.8)	0.389	0.057

FatMax = maximum fat oxidation zone; HR = heart rate; RER = respiratory exchange ratio; %VO_2peak_ = VO_2_R_peak_ = relative maximum oxygen consumption; V = velocity; AbsPw = absolute power; Pw/BM = relative to body weight power; RE = running Economy; L0 = 0% body mass load, L5 = 5% body mass load; L10 = 10% body mass load; *p* = ANOVA between-group differences.

**Table 5:** Differences in blood lactate, pH, and SO_2_ with time factor (pre and post) and group factor (L0, L5, and L10).

Variables	L0	L5	L10	*p*	ηp2
Lactate, pre (mmol/L)	1.86 (0.50)	2.01 (0.67)	1.98 (0.65)	0.309	0.071
Lactate, post (mmol/L)	11.48 (2.20)	11.58 (1.60)	10.99 (1.90)		
pH, pre	7.40 (0.02)	7.40 (0.03)	7.40 (0.02)	0.083 #	0.144
pH, post	7.24 (0.04)	7.25 (0.05)	7.26 (0.05)		
SO_2_, pre (%)	92.59 (2.90)	93.30 (2.69)	93.39 (1.59)	0.785	0.015
SO_2_, post (%)	93.91 (1.26)	94.10 (1.56)	94.37 (1.56)		
Step phase RPE	7.35 (0.81)	7.71 (1.02)	7.91 (0.81)	0.064	0.158
Ramp phase RPE	9.44 (0.58)	9.47 (0.54)	9.62 (0.42)	0.267	0.079

SO_2_= oxygen saturation; RPE = rate of perceived exertion; L0 = 0% body mass load, L5 = 5% body mass load; L10 = 10% body mass load. # = trend toward statistical significance in ANOVA interaction. ηp2 = partial eta square; *p* = ANOVA between-group differences.

## Data Availability

The original data report is available to reviewers by contacting the corresponding author.

## References

[1] Urbaneja J.S., Inés Farias E. (2017). El trail running (carreras de o por montaña) en España. Inicios, evolución y (actual) estado de la situación (Trail running in Spain. Origin, evolution and current situation; natural áreas). Retos Nuevas Perspect. Educ. Fís. Deporte Recreación.

[2] itra.run WMTRC: World Mountain & Trail Running Championship 2022. https://itra.run/Races/RaceResults/World.Mountain...Trail.Running.Championship.Long.Trail.80K/2022/79126.

[3] International Skyrunning Federatio General Assembly International Skyrunning Federation Rules. https://www.skyrunning.com/rules/.

[4] Lemire M., Hureau T.J., Favret F., Geny B., Kouassi B.Y.L., Boukhari M., Lonsdorfer E., Remetter R., Dufour S.P. (2021). Physiological factors determining downhill vs. uphill running endurance performance. J. Sci. Med. Sport.

[5] Sabater-Pastor F., Tomazin K., Millet G.P., Verney J., Féasson L., Millet G.Y. (2023). VO_2max_ and Velocity at VO_2max_ Play a Role in Ultradistance Trail-Running Performance. Int. J. Sports Physiol. Perform..

[6] Scheer V., Vieluf S., Janssen T.I., Heitkamp H. (2019). Predicting Competition Performance in Short Trail Running Races with Lactate Thresholds. J. Hum. Kinet..

[7] Alvero-Cruz J.R., Parent Mathias V., Garcia Romero J., Carrillo De Albornoz-Gil M., Benítez-Porres J., Ordoñez F.J., Rosemann T., Nikolaidis P.T., Knechtle B. (2019). Prediction of Performance in a Short Trail Running Race: The Role of Body Composition. Front. Physiol..

[8] Ehrström S., Tartaruga M.P., Easthope C.S., Brisswalter J., Morin J.B., Vercruyssen F. (2018). Short Trail Running Race: Beyond the Classic Model for Endurance Running Performance. Med. Sci. Sports Exerc..

[9] Lemire M., Falbriard M., Aminian K., Millet G.P., Meyer F. (2021). Level, Uphill, and Downhill Running Economy Values Are Correlated Except on Steep Slopes. Front. Physiol..

[10] Balducci P., Clémençon M., Trama R., Blache Y., Hautier C. (2017). Performance Factors in a Mountain Ultramarathon. Int. J. Sports Med..

[11] Vernillo G., Giandolini M., Edwards W.B., Morin J.B., Samozino P., Horvais N., Millet G.Y. (2017). Biomechanics and Physiology of Uphill and Downhill Running. Sports Med..

[12] Giandolini M., Pavailler S., Samozino P., Morin J.B., Horvais N. (2015). Foot strike pattern and impact continuous measurements during a trail running race: Proof of concept in a world-class athlete. Footwear Sci..

[13] Giandolini M., Horvais N., Rossi J., Millet G.Y., Morin J.B., Samozino P. (2016). Acute and delayed peripheral and central neuromuscular alterations induced by a short and intense downhill trail run: Fatigue after a downhill trail run. Scand. J. Med. Sci. Sports..

[14] Easthope C.S., Hausswirth C., Louis J., Lepers R., Vercruyssen F., Brisswalter J. (2010). Effects of a trail running competition on muscular performance and efficiency in well-trained young and master athletes. Eur. J. Appl. Physiol..

[15] Varesco G., Coratella G., Rozand V., Cuinet B., Lombardi G., Mourot L., Vernillo G. (2022). Downhill running affects the late but not the early phase of the rate of force development. Eur. J. Appl. Physiol..

[16] Lemire M., Hureau T.J., Remetter R., Geny B., Kouassi B.Y.L., Lonsdorfer E., Horobeti M.E.I., Favret F., Dufour S.P. (2020). Trail Runners Cannot Reach VO_2max_ during a Maximal Incremental Downhill Test. Med. Sci. Sports Exerc..

[17] Lemire M., Remetter R., Hureau T.J., Kouassi B.Y.L., Lonsdorfer E., Geny B., Horobeti M.E.I., Favret F., Dufour S.P. (2021). High-intensity downhill running exacerbates heart rate and muscular fatigue in trail runners. J. Sports Sci..

[18] Rojas-Valverde D., Sánchez-Ureña B., Pino-Ortega J., Gómez-Carmona C., Gutiérrez-Vargas R., Timón R., Olcina G. (2019). External Workload Indicators of Muscle and Kidney Mechanical Injury in Endurance Trail Running. Int. J. Environ. Res. Public Health.

[19] Gutiérrez-Vargas R., Martín-Rodríguez S., Sánchez-Ureña B., Rodríguez-Montero A., Salas-Cabrera J., Gutiérrez-Vargas J.C., Simunic B., Rojas-Valverde D. (2020). Biochemical and Muscle Mechanical Postmarathon Changes in Hot and Humid Conditions. J. Strength Cond. Res..

[20] Costa R.J.S., Knechtle B., Tarnopolsky M., Hoffman M.D. (2019). Nutrition for Ultramarathon Running: Trail, Track, and Road. Int. J. Sport Nutr. Exerc. Metab..

[21] Alcaraz P.E., Carlos-Vivas J., Oponjuru B.O., Martínez-Rodríguez A. (2018). The Effectiveness of Resisted Sled Training (RST) for Sprint Performance: A Systematic Review and Meta-analysis. Sports Med..

[22] Alcaraz P.E., Palao J.M., Elvira J.L.L., Linthorne N.P. (2008). Effects of Three Types of Resisted Sprint Training Devices on the Kinematics of Sprinting at Maximum Velocity. J. Strength Cond. Res..

[23] Carlos-Vivas J., Marín-Cascales E., Freitas T.T., Perez-Gomez J., Alcaraz P.E. (2019). Force-Velocity-Power Profiling During Weighted-Vest Sprinting in Soccer. Int. J. Sports Physiol. Perform..

[24] Clark K.P., Stearne D.J., Walts C.T., Miller A.D. (2010). The Longitudinal Effects of Resisted Sprint Training Using Weighted Sleds vs. Weighted Vests. J. Strength Cond. Res..

[25] Cross M.R., Brughelli M.E., Cronin J.B. (2014). Effects of Vest Loading on Sprint Kinetics and Kinematics. J. Strength Cond. Res..

[26] Carretero-Navarro G., Márquez G., Cherubini D., Taube W. (2019). Effect of different loading conditions on running mechanics at different velocities. Eur. J. Sport Sci..

[27] Gaffney C.J., Cunnington J., Rattley K., Wrench E., Dyche C., Bampouras T.M. (2022). Weighted vests in CrossFit increase physiological stress during walking and running without changes in spatiotemporal gait parameters. Ergonomics.

[28] Moore K.J., Penry J.T., Gunter K.B. (2014). Development of a Walking Aerobic Capacity Test for Structural Firefighters. J. Strength Cond. Res..

[29] Klimek A.T., Klimek A. (2007). The weighted walking test as an alternative method of assessing aerobic power. J. Sports Sci..

[30] Purdom T.M., Mermier C., Dokladny K., Moriarty T., Lunsford L., Cole N., Johnson K., Kravitz L. (2019). Predictors of Fat Oxidation and Caloric Expenditure With and Without Weighted Vest Running. J. Strength Cond. Res..

[31] Puthoff M.L., Darter B.J., Nielsen D.H., Yack H.J. (2006). The effect of weighted vest walking on metabolic responses and ground reaction forces. Med. Sci. Sports Exerc..

[32] World Medical Association (2013). World Medical Association Declaration of Helsinki: Ethical Principles for Medical Research Involving Human Subjects. JAMA.

[33] Norton K.I., Norton K., Eston R. (2018). Standards for Anthropometry Assessment. Kinanthropometry and Exercise Physiology.

[34] Dimitrijevic M., Lalovic D., Milovanov D. (2021). Correlation of Different Anthropometric Methods and Bioelectric Impedance in Assessing Body Fat Percentage of Professional Male Athletes. Serbian J. Exp. Clin. Res..

[35] Urbaniak G.C., Plous S. (2013). Research Randomizer.

[36] Zuniga J.M., Housh T.J., Camic C.L., Bergstrom H.C., Traylor D.A., Schmidt R.J., Johnson G.O. (2012). Metabolic parameters for ramp versus step incremental cycle ergometer tests. Appl. Physiol. Nutr. Metab..

[37] Larson R.D., Cantrell G.S., Ade C.J., Farrell J.W., Lantis D.J., Barton M.A., Larson D.J. (2015). Physiologic Responses to Two Distinct Maximal Cardiorespiratory Exercise Protocols. Int. J. Sports Exerc. Med..

[38] Binder R.K., Wonisch M., Corra U., Cohen-Solal A., Vanhees L., Saner H., Schmid J.-P. (2008). Methodological approach to the first and second lactate threshold in incremental cardiopulmonary exercise testing. Eur. J. Cardiovasc. Prev. Rehabil..

[39] Scherr J., Wolfarth B., Christle J.W., Pressler A., Wagenpfeil S., Halle M. (2013). Associations between Borg’s rating of perceived exertion and physiological measures of exercise intensity. Eur. J. Appl. Physiol..

[40] Mier C.M., Alexander R.P., Mageean A.L. (2012). Achievement of VO2max Criteria During a Continuous Graded Exercise Test and a Verification Stage Performed by College Athletes. J. Strength Cond. Res..

[41] Wasserman K., Whipp B.J., Koyl S.N., Beaver W.L. (1973). Anaerobic threshold and respiratory gas exchange during exercise. J. Appl. Physiol..

[42] Frayn K.N. (1983). Calculation of substrate oxidation rates in vivo from gaseous exchange. J. Appl. Physiol..

[43] Poole D.C., Wilkerson D.P., Jones A.M. (2008). Validity of criteria for establishing maximal O_2_ uptake during ramp exercise tests. Eur. J. Appl. Physiol..

[44] Cohen J. (2009). Statistical Power Analysis for the Behavioral Sciences.

[45] Barnes K.R., Hopkins W.G., McGuigan M.R., Kilding A.E. (2015). Warm-up with a weighted vest improves running performance via leg stiffness and running economy. J. Sci. Med. Sport..

[46] Cartón-Llorente A., Rubio-Peirotén A., Cardiel-Sánchez S., Roche-Seruendo L.E., Jaén-Carrillo D. (2023). Training Specificity in Trail Running: A Single-Arm Trial on the Influence of Weighted Vest on Power and Kinematics in Trained Trail Runners. Sensors.

